# Association between iodine nutrition and cervical lymph node metastasis of papillary thyroid microcarcinoma

**DOI:** 10.3389/fendo.2023.1164069

**Published:** 2023-08-31

**Authors:** Hengqiang Zhao, Jin Hu, Le Cui, Yiping Gong, Tao Huang

**Affiliations:** ^1^ Department of Breast and Thyroid Surgery, Renmin Hospital of Wuhan University, Wuhan, China; ^2^ Department of Breast and Thyroid Surgery, Union Hospital, Tongji Medical College, Huazhong University of Science and Technology, Wuhan, China

**Keywords:** papillary thyroid microcarcinoma, iodine, iodine nutrition, urinary iodine concentration, lymph node metastasis

## Abstract

We aimed to investigate the association between iodine intake and nodal metastasis stratified by central lymph node metastasis (CLNM) and lateral lymph node metastasis (LLNM) of papillary thyroid microcarcinoma (PTMC). Urinary iodine concentration (UIC) and clinicopathological characteristics were used to identify factors associated with CLNM and LLNM using logistic regression analysis. A sum of 3,858 PTMC patients were enrolled. The median UIC (MUI) of patients with CLNM or LLNM was not statistically different from those without nodal metastasis. Male patients had a higher MUI than females (183.4 μg/L vs. 173.6 μg/L). Female patients with extracapsular extension had a higher MUI than those without it (210.0 μg/L vs. 172.1 μg/L). Male patients with LLNM had a significantly lower MUI than those without LLNM (134.7 μg/L vs. 187.9 μg/L). Female patients with more than adequate iodine intake were more likely to present with CLNM and extrathyroidal extension than those with adequate iodine intake with an odds ratio (95% confidence interval) of 1.23 (1.01–1.51) and 1.59 (1.09–2.32) after adjustment. Iodine nutrition was not found to be associated with LLNM. In addition, patients with a younger age, larger tumors, extrathyroidal extension, and intrathyroidal spread were more likely to be CLNM, whereas nodular goiter presented with a protective factor; CLNM was the only factor associated with LLNM of PTMC in both genders. In conclusion, iodine nutrition has a much closer association with female than male patients, and high iodine intake may be associated with CLNM and extrathyroidal extension in female PTMC patients.

## Introduction

The overall incidence of thyroid cancer increased by 3% annually in the United States from 1974 to 2013 (1). In China, the age-standardized incidence of thyroid cancer was 3.21/10^5^ in 2005 and 9.61/10^5^ in 2015 (2). The increasing incidence of thyroid cancer is due in large part to the epidemiology of surveillance and overdiagnosis but that there also appears to be a true increase in new cases of thyroid cancer (3). The increased incidence mainly attributes to papillary thyroid cancer (PTC), especially papillary thyroid microcarcinoma (PTMC) with its maximum tumor diameter ≤1 cm (4).

Iodine is an indispensable element for synthesis of thyroxine and is closely associated with thyroid diseases (5). High iodine intake was associated with the occurrence of Hashimoto thyroiditis, nodular goiter, and hyperthyroidism (6). However, the association between iodine intake and thyroid cancer remains controversial (7). Some studies reported that mild iodine deficiency may contribute to the exceptionally high incidence of thyroid cancer (8), whereas others reported that excessive iodine nutrition was associated with PTC and PTMC occurrence compared with adequate iodine intake (9, 10). We previously found that high iodine intake was not associated with PTC incidence in the general population and in patients with thyroid nodules (11). However, more than adequate iodine intake was independently associated with a larger tumor size of PTC patients (11). Thus, we expected that high iodine intake may be associated with the progression of PTC.

Central lymph node metastasis (CLNM) was not uncommon in PTMC patients with its incidence up to 51.7% (12). In addition, approximately 4.4% of patients with PTMC presented with lateral lymph node metastasis (LLNM) at presentation (13). Accumulating evidence suggested that cervical lymph node metastasis was a risk factor for PTC recurrence (14). Some scholars hold that low iodine intake was inversely associated with CLNM of PTC, particularly for PTMC (15). However, others reported that high iodine nutrition seemed to be associated with CLNM of PTC (16). We previously found that excessive iodine intake was marginally associated with CLNM in female PTC patients after defining CLNM as metastatic lymph nodes ≥2 (12). However, we failed to investigate the effect of iodine nutrition on PTMC progression.

Considering the limited and inconsistent results, we aimed to comprehensively investigate the association between iodine nutrition and clinicopathologic characteristics of PTMC. We divided patients into CLNM and LLNM subgroups and explored their associations with urinary iodine concentration (UIC). This study may provide new evidence of the effect of iodine nutrition on PTMC progression.

## Patients and methods

### Study population

Patients with PTMC were retrospectively enrolled from 2013 to 2018 in Union hospital, Wuhan, China. This study was approved by the Ethics Committee of Union hospital. The enrolled patients underwent thyroidectomy with central lymph node dissection (CLND), and lateral lymph nodes dissection (LLND) or not for the first time. CLND was routinely performed on PTMC patients. LLND was performed based on preoperative imaging suspicious for malignancy and/or fine-needle aspiration cytology preoperatively. Solitary focus means only one tumor in the thyroid, whereas multiple foci mean two or more foci limited to the thyroid. Bilaterality is defined as the presence of PTC foci in the left and right lobes of the thyroid. Capsular invasion means that one tumor invades the thyroid capsule but does not penetrate it, whereas one tumor penetrates the capsule into the strap muscle or perithyroidal fibrofatty tissues, which will be defined as extracapsular or extrathyroidal extension. Intrathyroidal spread means that the primary tumor spreads to the other parts of the thyroid through intrathyroidal lymph vessels. Intraoperative frozen section was routinely performed to determine thyroid malignancy. The final pathological types, tumor number, intrathyroidal spread, capsular invasion and extrathyroidal extension, central lymph node metastasis (CLNM), and lateral lymph node metastasis (LLNM) were determined by postoperative specimen. Hematoxylin and eosin staining with or without immunohistochemistry was performed to determine the pathology or nodal metastasis of PTMC. The pathological types included PTC, PTC and nodular goiter, PTC and Hashimoto’s thyroiditis, and PTC and nodular goiter, and Hashimoto’s thyroiditis. Patients with iodine pretreatment for hyperthyroidism, pathology other than PTC, and reoperation for PTC were excluded.

### Iodine nutritional status

Every patient was asked to collect a fasting urine specimen (6). Urinary iodine concentration (UIC) was measured using Direction of Quantitative Test Kit type AR for urinary iodine (Wuhan, China) as previously described (11). Median UIC (MUI) was recommended to assess iodine nutritional status in population according to World Health Organization, and iodine nutritional status was divided into four categories: iodine deficiency (UIC <100 μg/L), adequate iodine intake (UIC: 100–199 μg/L), more than adequate iodine intake (UIC: 200–299 μg/L), and excessive iodine intake (UIC ≥300 μg/L).

### Statistical analysis

Variables with skewed distribution were expressed as median (upper and lower quartile) and analyzed using the Mann–Whitney *U* test. Categorical data were analyzed with the chi-square test. Multivariate logistic regression analysis was performed to identify factors associated with nodal metastasis. All the analyses were performed using SPSS 22.0 software. A two-sided *P* value <0.05 was considered significant.

## Results

### The iodine nutrition of PTMC patients

**Figure 1:**
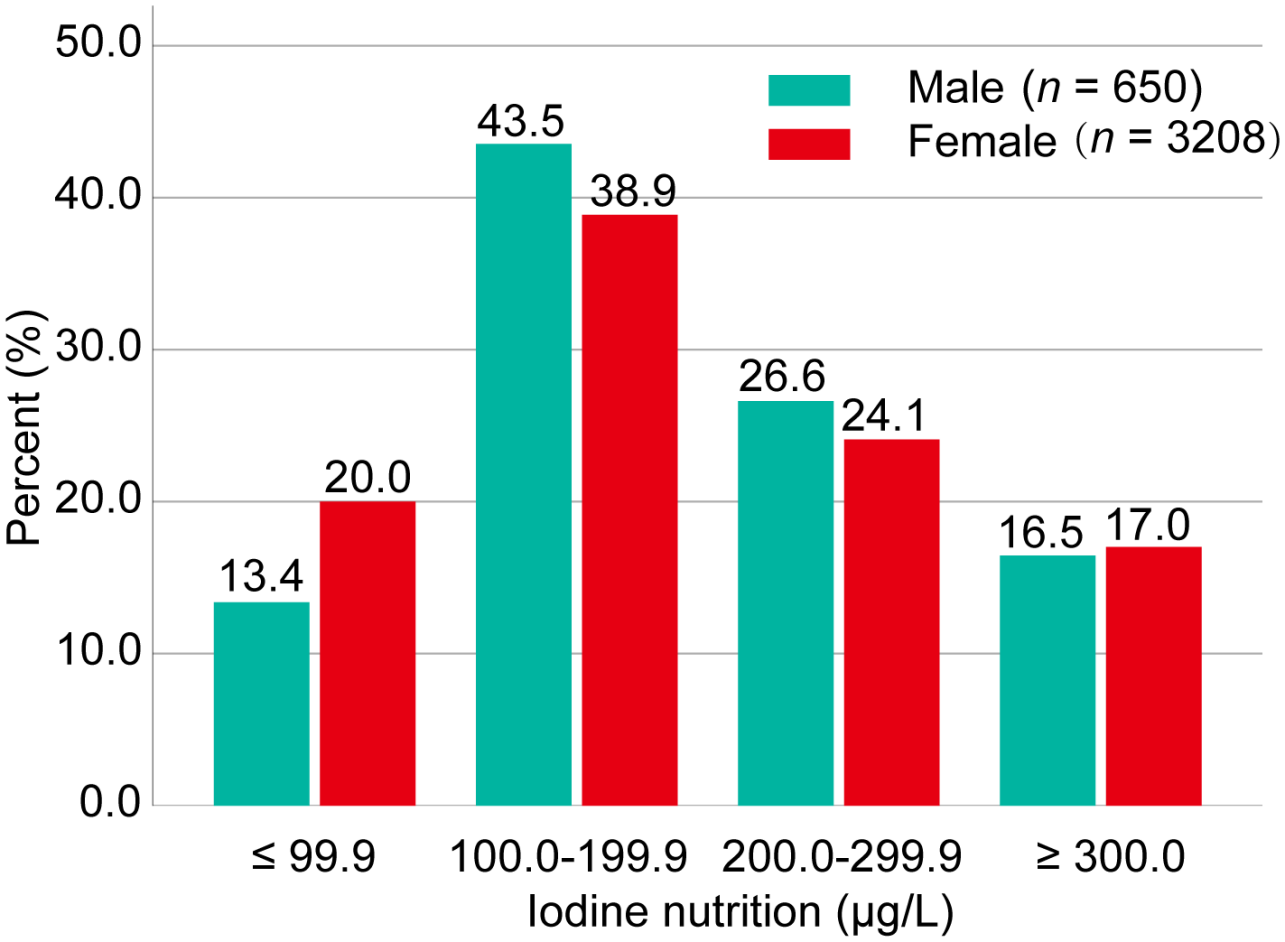
Iodine status of the study population. Iodine nutrition was classified into four categories: iodine deficiency (UIC <100.0 μg/L), adequate iodine intake (UIC: 100.0–199.9 μg/L), more than adequate iodine intake (UIC: 200.0–299.9), and excessive iodine intake (UIC ≥300.0 μg/L). Data were expressed as percentage (%). UIC, urinary iodine concentration.

Of the 3,858 patients enrolled, iodine deficiency was more common in female than male PTMC patients (20.0% vs. 13.4%). Adequate iodine intake accounted for 39.7% of the total population. More than adequate and excessive iodine intake accounted for 41.4% (**Figure 1**).

The MUI was significantly higher in males than in females (MUI: 183.4 μg/L vs. 173.6 μg/L; *P* = 0.038). In addition, patients with extrathyroidal extension had a significantly higher MUI than those without it (MUI: 199.7 μg/L vs. 174.3 μg/L; *P* = 0.024). Patients with a younger age (<55 years) and intrathyroidal spread had a marginally higher MUI than the corresponding counterparts. The MUI in the CLNM and non-CLNM groups was 177.9 μg/L and 173.9 μg/L, respectively (*P* = 0.554). The MUI in the LLNM and non-LLNM groups of PTMC patients was 157.9 μg/L and 176.5 μg/L, respectively (*P* = 0.132) (**Table 1**).

**Table 1:** Comparison of MUI in PTMC patients (*n* = 3,858).

Variables	*n*	MUI (μg/L)	*P*
Sex			
Male	650	183.4 (127.1–253.8)	0.038
Female	3,208	173.6 (111.4–262.9)	
Age (year)			
<55	3,175	177.2 (117.1–263.4)	0.058
≥55	683	165.9 (108.7–250.3)	
Tumor size (cm)			
D ≤ 0.5	1,996	173.1 (114.6–260.8)	0.387
0.5 < D ≤ 1.0	1,862	177.5 (115.8–261.9)	
Tumor number			
Solitary	2,208	178.2 (117.4–257.9)	0.818
Multiple	1,650	171.5 (112.7–265.5)	
Bilaterality			
No	2,711	178.2 (117.8–260.6)	0.475
Yes	1,147	167.4 (110.2–262.1)	
Capsular invasion			
No	1,878	172.5 (111.3–262.8)	0.366
Yes	1,980	178.2 (117.8–259.3)	
Extracapsular extension			
No	3,620	174.3 (114.5–260.8)	0.024
Yes	238	199.7 (130.0–269.8)	
Intrathyroidal spread			
No	3,744	174.7 (114.5–260.8)	0.076
Yes	114	203.7 (132.9–277.2)	
CLNM			
No	2,341	173.9 (114.5–263.1)	0.544
Yes	1,517	177.9 (116.0–257.2)	
LLNM			
No	3,623	176.5 (115.2–262.1)	0.132
Yes	235	157.9 (108.5–236.8)	
Pathology			
PTMC	1,122	179.8 (124.7–257.5)	0.105
PTMC&HT&NG	520	166.1 (105.6–257.1)	
PTMC&HT	1,446	173.1 (107.3–269.6)	
PTMC&NG	770	176.5 (118.9–256.2)	

MUI, median urinary iodine; PTMC, papillary thyroid microcarcinoma; D, diameter; CLNM, central lymph node metastasis; LLNM, lateral lymph node metastasis; HT, Hashimoto’s thyroiditis; NG, nodular goiter.

We then evaluated the iodine nutrition in male and female PTMC patients, separately. We found that male patients with LLNM had a significantly lower MUI than those without LLNM (MUI: 134.7 μg/L vs. 187.9 μg/L; *P* = 0.002). In addition, female patients with extrathyroidal extension had a significantly higher MUI than those without it (MUI: 210.0 μg/L vs. 172.1 μg/L; *P* = 0.010) (**Table 2**).

**Table 2:** Comparison of MUI in PTMC patients stratified by gender (*n* = 3,858).

Variables	Male			Female		
	*n*	MUI (μg/L)	*P*	*n*	MUI (μg/L)	*P*
Age (year)						
<55	550	184.2 (132.0–255.2)	0.109	2625	175.3 (111.7–265.5)	0.163
≥55	100	176.6 (107.1–251.7)		583	164.5 (110.0–248.3)	
Tumor size (cm)						
D ≤ 0.5	301	180.9 (123.8–252.8)	0.846	1695	171.3 (111.7–263.4)	0.694
0.5 < D ≤ 1.0	349	184.6 (127.8–255.6)		1513	176.0 (111.1–262.9)	
Tumor number						
Solitary	391	184.5 (127.7–251.0)	0.994	1817	177.2 (112.0–261.0)	0.810
Multiple	259	181.8 (122.5–260.8)		1391	169.0 (110.0–265.5)	
Bilaterality						
No	476	183.7 (125.9–254.9)	0.706	2235	177.2 (115.1–262.4)	0.312
Yes	174	181.9 (129.9–253.0)		973	165.9 (106.6–265.5)	
Capsular invasion						
No	306	187.5 (121.1–255.2)	0.787	1572	168.9 (108.5–265.2)	0.314
Yes	344	180.3 (131.4–252.1)		1636	177.8 (114.4–261.9)	
Extracapsular extension						
No	597	184.0 (127.8–255.0)	0.745	3023	172.1 (110.8–262.4)	0.010
Yes	53	173.0 (120.8–245.5)		185	210.0 (131.8–282.1)	
Intrathyroidal spread						
No	624	184.2 (127.3–253.3)	0.967	3120	172.7 (111.1–262.9)	0.068
Yes	26	175.4 (126.3–294.6)		88	211.3 (137.9–275.5)	
CLNM						
No	320	192.7 (131.4–259.3)	0.099	2021	171.4 (111.3–263.6)	0.271
Yes	330	177.4 (122.1–247.3)		1187	178.2 (112.5–261.6)	
LLNM						
No	591	187.9 (132.2–255.2)	0.002	3032	173.8 (111.3–263.9)	0.789
Yes	59	134.7 (107.1–207.2)		176	166.1 (120.0–240.7)	
Pathology						
PTMC	336	193.1 (136.3–261.0)	0.066	786	174.7 (119.7–251.3)	0.399
PTMC&NG, HT	22	146.3 (115.5–233.9)		498	166.2 (104.9–259.6)	
PTMC&HT	148	167.4 (118.0–250.7)		1298	174.6 (106.5–272.0)	
PTMC&NG	144	178.7 (119.8–251.9)		626	175.6 (118.6–257.3)	

MUI, median urinary iodine; PTMC, papillary thyroid microcarcinoma; D, diameter; CLNM, central lymph node metastasis; LLNM, lateral lymph node metastasis; HT, Hashimoto’s thyroiditis; NG, nodular goiter.

We divided UIC into four categories: iodine deficiency, adequate iodine intake, more than adequate iodine intake, and excessive iodine intake. Similarly, we found that iodine nutrition status was not significantly different between patients with CLNM or LLNM and those without nodal metastasis (**Supplementary Table 1**). In addition, males were more likely to be adequate iodine intake and more than adequate iodine intake compared with females (70.1% vs. 63.0%). Additionally, patients with extrathyroidal extension were more likely to be more than adequate iodine intake and excessive iodine intake than those without it (50% vs. 40.9%) (**Supplementary Table 1**).

### Association between iodine intake and CLNM in male PTMC

In the univariate analysis, iodine deficiency and more than adequate and excessive iodine intake were not associated with CLNM compared with adequate iodine intake. After adjustment, iodine nutrition status was not found to be associated with CLNM in male PTMC patients. In the multivariate model, a younger age, larger tumor size, extrathyroidal extension, intrathyroidal spread, and LLNM were associated with CLNM. Of note, PTMC patients accompanied by nodular goiter were inversely associated with CLNM compared with PTMC alone (**Table 3**).

**Table 3:** Association between iodine intake and CLNM in male PTMC patients (*n* = 650).

Variables	Univariate		Multivariate	
	OR (95% CI)	*P*	OR (95% CI)	*P*
Age (year)				
<55	Ref		Ref	
≥55	0.40 (0.25–0.63)	<0.001	0.45 (0.27–0.77)	0.003
Tumor size (cm)				
D ≤ 0.5	Ref		Ref	
0.5 < D ≤ 1.0	1.91 (1.39–2.60)	<0.001	1.55 (1.09–2.21)	0.016
Tumor number				
Solitary	Ref		Ref	
Multiple	1.45 (1.06–1.99)	0.020	1.29 (0.79–2.11)	0.308
Bilaterality				
No	Ref		Ref	
Yes	1.45 (1.02–2.05)	0.039	1.15 (0.67–1.98)	0.609
Capsular invasion				
No	Ref		Ref	
Yes	1.62 (1.19–2.21)	0.002	1.07 (0.75–1.53)	0.697
Extracapsular extension				
No	Ref		Ref	
Yes	2.93 (1.56–5.51)	0.001	2.65 (1.30–5.38)	0.007
Intrathyroidal spread				
No	Ref		Ref	
Yes	5.64 (1.92–16.56)	0.002	4.83 (1.52–15.35)	0.008
LLNM				
No	Ref		Ref	
Yes	10.01 (4.24–23.65)	<0.001	10.22 (4.02–25.94)	< 0.001
Pathology				
PTMC	Ref		Ref	
PTMC&NG&HT	0.64 (0.27–1.52)	0.313	0.38 (0.12–1.15)	0.087
PTMC&HT	0.96 (0.65–1.41)	0.815	0.85 (0.56–1.29)	0.437
PTMC&NG	0.38 (0.26–0.58)	<0.001	0.47 (0.30–0.74)	0.001
UIC (μg/L)				
≤99.9	1.16 (0.72–1.88)	0.548	1.21 (0.70–2.07)	0.495
100.0–199.9	Ref		Ref	
200.0–299.9	0.81 (0.56–1.18)	0.277	0.86 (0.57–1.29)	0.460
≥300.0	0.79 (0.51–1.23)	0.297	0.72 (0.44–1.18)	0.195

CLNM, central lymph node metastasis; PTMC, papillary thyroid microcarcinoma; OR (95% CI), odds ratio (95% confidence interval); D, diameter; LLNM, lateral lymph node metastasis; HT, Hashimoto’s thyroiditis; NG, nodular goiter; UIC, urinary iodine concentration.

### Association between iodine intake and CLNM in female PTMC

In the univariate analysis, more than adequate iodine intake was associated with CLNM compared with adequate iodine intake with OR (95% CI) of 1.25 (1.04–1.50) in female PTMC patients. After adjustment, patients with more than adequate iodine intake were associated with CLNM than those with adequate iodine intake with OR (95% CI) of 1.23 (1.01–1.51). In addition, a younger age, lager tumor size, bilaterality, capsular invasion, intrathyroidal spread, and LLNM were positively associated with CLNM in female PTMC after adjustment. Of note, PTMC patients accompanied by nodular goiter, or nodular goiter and Hashimoto’s thyroiditis were inversely associated with CLNM compared with PTMC alone (**Table 4**).

**Table 4:** Association between iodine intake and CLNM in female PTMC patients (*n* = 3208).

Variables	Univariate		Multivariate	
	OR (95% CI)	*P*	OR (95% CI)	*P*
Age (year)				
<55	Ref		Ref	
≥55	0.47 (0.38–0.58)	<0.001	0.50 (0.41–0.63)	<0.001
Tumor size (cm)				
D ≤ 0.5	Ref		Ref	
0.5 <D ≤ 1.0	2.58 (2.23–2.99)	<0.001	1.91 (1.62–2.24)	<0.001
Tumor number				
Solitary	Ref		Ref	
Multiple	1.74 (1.51–2.02)	<0.001	1.15 (0.91–1.45)	0.255
Bilaterality				
No	Ref		Ref	
Yes	1.97 (1.69–2.29)	<0.001	1.48 (1.15–1.90)	0.002
Capsular invasion				
No	Ref		Ref	
Yes	2.02 (1.75–2.34)	<0.001	1.41 (1.20–1.67)	<0.001
Extracapsular extension				
No	Ref		Ref	
Yes	1.91 (1.42–2.57)	<0.001	1.12 (0.80–1.57)	0.506
Intrathyroidal spread				
No	Ref		Ref	
Yes	5.03 (3.11–8.14)	<0.001	3.04 (1.80–5.14)	<0.001
LLNM				
No	Ref		Ref	
Yes	12.85 (8.24–20.05)	<0.001	8.56 (5.41–13.56)	<0.001
Pathology				
PTMC	Ref		Ref	
PTMC&NG&HT	0.49 (0.38–0.62)	<0.001	0.61 (0.47–0.79)	<0.001
PTMC&HT	0.98 (0.82–1.17)	0.819	0.97 (0.80–1.17)	0.735
PTMC&NG	0.58 (0.47–0.73)	<0.001	0.71 (0.56–0.90)	0.005
UIC (μg/L)				
≤99.9	1.02 (0.84–1.25)	0.816	1.03 (0.83–1.28)	0.775
100.0–199.9	Ref		Ref	
200.0–299.9	1.25 (1.04–1.50)	0.019	1.23 (1.01–1.51)	0.042
≥300.0	1.09 (0.89–1.35)	0.399	1.02 (0.81–1.28)	0.871

CLNM, central lymph node metastasis; PTMC, papillary thyroid microcarcinoma; OR (95% CI), odds ratio (95% confidence interval); D, diameter; LLNM, lateral lymph node metastasis; HT, Hashimoto’s thyroiditis; NG, nodular goiter; UIC, urinary iodine concentration.

### Association between iodine intake and LLNM in male PTMC

In the univariate analysis, more than adequate iodine intake was inversely associated with LLNM compared with adequate iodine intake with OR (95% CI) of 0.45 (0.21–0.96) in male PTMC patients. After adjustment, iodine nutrition status was not found to be associated with LLNM in male PTMC patients. In the multivariate model, results showed that multifocality, CLNM, and PTMC and nodular goiter, and Hashimoto’s thyroiditis were independently associated with LLNM in male PTMC patients (**Table 5**).

**Table 5:** Association between iodine intake and LLNM in male PTMC patients (*n* = 650).

Variables	Univariate		Multivariate	
	OR (95% CI)	*P*	OR (95% CI)	*P*
Age (year)				
<55	Ref		Ref	
≥55	0.99 (0.47–2.08)	0.977	1.14 (0.44–2.97)	0.786
Tumor size (cm)				
D ≤ 0.5	Ref		Ref	
0.5 <D ≤ 1.0	1.63 (0.93–2.84)	0.086	1.73 (0.88–3.40)	0.111
Tumor number				
Solitary	Ref		Ref	
Multiple	2.58 (1.49–4.47)	0.001	2.48 (1.12–5.47)	0.025
Bilaterality				
No	Ref		Ref	
Yes	1.86 (1.07–3.24)	0.028	0.77 (0.35–1.73)	0.530
Capsular invasion				
No	Ref		Ref	
Yes	1.56 (0.90–2.70)	0.116	1.08 (0.56–2.05)	0.827
Extracapsular extension				
No	Ref		Ref	
Yes	2.60 (1.23–5.49)	0.012	1.37 (0.57–3.30)	0.480
Intrathyroidal spread				
No	Ref		Ref	
Yes	3.23 (1.24–8.40)	0.016	2.18 (0.75–6.34)	0.153
CLNM				
No	Ref		Ref	
Yes	10.01 (4.24–23.65)	<0.001	9.23 (3.68–23.16)	<0.001
Pathology				
PTMC	Ref		Ref	
PTMC&NG&HT	7.11 (2.72–18.56)	<0.001	12.64 (3.48–45.90)	<0.001
PTMC&HT	1.51 (0.78–2.92)	0.222	1.44 (0.69–3.00)	0.328
PTMC&NG	0.93 (0.43–1.99)	0.848	1.30 (0.54–3.12)	0.564
UIC (μg/L)				
≤99.9	1.30 (0.64–2.66)	0.471	1.27 (0.57–2.82)	0.560
100.0–199.9	Ref		Ref	
200.0–299.9	0.45 (0.21–0.96)	0.039	0.57 (0.25–1.29)	0.177
≥300.0	0.57 (0.24–1.33)	0.195	0.57 (0.22–1.46)	0.237

LLNM, lateral lymph node metastasis; PTMC, papillary thyroid microcarcinoma; OR (95% CI), odds ratio (95% confidence interval); D, diameter; CLNM, central lymph node metastasis; HT, Hashimoto’s thyroiditis; NG, nodular goiter; UIC, urinary iodine concentration.

### Association between iodine intake and LLNM in female PTMC

Iodine nutrition status was not found to be associated with LLNM in female PTMC patients in the univariate and multivariate logistic regression analyses. After adjustment, larger tumor size, extrathyroidal extension, intrathyroidal spread, CLNM, and PTMC and Hashimoto’s thyroiditis were independently associated with LLNM in female PTMC patients (**Table 6**).

**Table 6:** Association between iodine intake and LLNM in female PTMC patients (*n* = 3208).

Variables	Univariate		Multivariate	
	OR (95% CI)	*P*	OR (95% CI)	*P*
Age (year)				
<55	Ref		Ref	
≥55	0.63 (0.40–0.99)	0.046	0.93 (0.56–1.53)	0.774
Tumor size (cm)				
D ≤ 0.5	Ref		Ref	
0.5 < D ≤ 1.0	5.06 (3.46–7.41)	<0.001	2.83 (1.89–4.24)	<0.001
Tumor number				
Solitary	Ref		Ref	
Multiple	2.11 (1.55–2.88)	<0.001	1.21 (0.73–2.02)	0.459
Bilaterality				
No	Ref		Ref	
Yes	2.20 (1.62–2.99)	<0.001	1.18 (0.71–1.95)	0.521
Capsular invasion				
No	Ref		Ref	
Yes	2.62 (1.87–3.67)	<0.001	1.14 (0.78–1.68)	0.490
Extracapsular extension				
No	Ref		Ref	
Yes	3.64 (2.37–5.59)	<0.001	2.47 (1.50–4.05)	<0.001
Intrathyroidal spread				
No	Ref		Ref	
Yes	6.42 (3.86–10.68)	<0.001	3.37 (1.90–5.96)	<0.001
CLNM				
No	Ref		Ref	
Yes	12.85 (8.24–20.05)	<0.001	8.47 (5.35–13.41)	<0.001
Pathology				
PTMC	Ref		Ref	
PTMC&NG&HT	0.28 (0.13–0.61)	0.001	0.51 (0.23–1.12)	0.093
PTMC&HT	1.41 (0.98–2.04)	0.068	1.64 (1.10–2.45)	0.015
PTMC&NG	0.78 (0.48–1.28)	0.321	1.21 (0.71–2.06)	0.486
UIC (μg/L)				
≤ 99.9	0.91 (0.60–1.39)	0.668	0.83 (0.53–1.31)	0.427
100.0–199.9	Ref		Ref	
200.0–299.9	0.99 (0.67–1.46)	0.962	0.76 (0.50–1.16)	0.202
≥300.0	0.98 (0.63–1.52)	0.920	0.78 (0.49–1.25)	0.295

LLNM, lateral lymph node metastasis; PTMC, papillary thyroid microcarcinoma; OR (95% CI), odds ratio (95% confidence interval); D, diameter; CLNM, central lymph node metastasis; HT, Hashimoto’s thyroiditis; NG, nodular goiter; UIC, urinary iodine concentration.

### Association between iodine nutrition and extrathyroidal extension in PTMC patients

In the univariate analysis, more than adequate iodine intake was associated with extrathyroidal extension with OR (95% CI) of 1.70 (1.18–2.44) in female PTMC patients. After adjustment, more than adequate iodine intake predicted a higher possibility for extrathyroidal extension with OR (95% CI) of 1.59 (1.09–2.32) compared with adequate iodine intake (**Supplementary Table 2**).

## Discussion

In the present study, we comprehensively investigated the association between iodine intake and nodal metastasis of PTMC patients for the first time. The present evidence suggested that more than adequate iodine intake was associated with CLNM and extrathyroidal extension compared with adequate iodine intake in female PTMC patients. Iodine nutrition was not found to be associated with CLNM in males and was not associated with LLNM in either gender.

The association between iodine intake and PTC has been controversial. Some hold that a higher exposure to iodine intake was associated with PTC incidence (9, 10, 17–21). However, some scholars hold that high iodine intake was not associated with PTC occurrence and high UIC was just a specific characteristic of the disease (22). Fan et al. reported that the areas with a low consumption rate of qualified iodized salt and areas with a high incidence of thyroid goiter had a relatively high incidence of thyroid cancer (8). We previously found that the MUI was not significantly different between patients with benign thyroid nodules and PTC, which was consistent with other reports (23). However, more than adequate iodine intake might be associated with the growth of PTC (≥1 cm vs. <1 cm) compared with adequate iodine intake (11). We thus expected that a high iodine intake might be associated with the progression of PTC.

Extrathyroidal extension and intrathyroidal spread were aggressive features of PTC. Huang et al. found that excessive iodine exposure was associated with capsular invasion and extrathyroidal metastases of PTC (19). Similarly, we previously reported that more than adequate and excessive iodine intake were marginally associated with capsular invasion of PTC (12). In this study, the MUI of patients with extrathyroidal extension and intrathyroidal spread was higher than that in the corresponding patients. In addition, more than adequate iodine intake was associated with extrathyroidal extension of PTMC. Accumulating evidence suggested that extrathyroidal extension and intrathyroidal spread were positively associated with CLNM of PTC (24, 25). We can expect that high iodine intake may induce CLNM *via* promoting the aggressiveness of PTMC. High iodine intake within a certain concentration may promote PTC cell proliferation *via* upregulating growth signaling pathways (26). However, the potential mechanisms remain explored *in vivo*.

Cervical nodal metastasis was common in PTC, as well as in PTMC (12). However, the available data failed to find a definitive association between iodine take and CLNM. Fan et al. reported that the proportions of UIC >300 μg/L and of serum iodine concentration >90 μg/L were higher in the CLNM (*n* = 167) than that in the non-CLNM (*n* = 235) PTC patients (16). However, we did not find a statistical difference in MUI between CLNM (*n* = 1517) and non-CLNM (*n* = 2341) patients with PTMC. Similarly, Huang et al. found that iodine nutrition was not associated with CLNM with a sum of 97 PTC patients *via* UIC determination (19). We found that more than adequate iodine intake was associated with CLNM, but not excessive iodine intake. In our opinion, it may be easier to be more than adequate iodine status than excessive iodine intake. The recommended intake of iodine for adults is 150 μg/day, and the maximum tolerable iodine intake is 1,000 μg/day. In addition, the high iodine intake status will take a long time to produce adverse effects. The occasionally excessive iodine intake may not cause adverse results. Therefore, studies on the relationship between excessive iodine intake like high iodine area and thyroid cancer should be investigated. We found that patients with LLNM (*n* = 235) had a lower MUI than those without LLNM for the first time, whereas iodine intake was not found to be associated with LLNM, which should be validated in the following studies.

The MUI of male patients was significantly higher than that in female patients. In general, women’s demand for iodine is greater than men’s. This will lead to a higher proportion of women with iodine deficiency than men (11), which was consistent with the present result. However, we found that high iodine intake was associated with extrathyroidal extension and CLNM in women, but not in men. This indicated that women were more vulnerable to iodine nutrition than men. A recent study revealed that more than adequate and excessive iodine intake had an inverse relationship with thyroid peroxidase antibody, and they were predictors for elevated thyrotropin (27). Furthermore, the higher thyrotropin level was associated with extrathyroidal extension and cervical lymph node metastasis of PTMC (28). Therefore, we speculated that high iodine intake might induce thyroid antibody production, leading to elevated thyrotropin levels and contributing to the progression of thyroid cancer. In addition, the average serum thyrotropin concentration and prevalence of antithyroid antibodies are greater in women relative to men in the general population (29). This suggested that thyroid disease should be considered during routine evaluation of this susceptible population and should be followed by appropriate detection and treatment.

In this study, PTMC patients accompanied by nodular goiter (NG) were inversely associated with CLNM compared with PTMC alone in both men and women. Similarly, some reported that PTC patients with NG had a lower incidence of CLNM compared with those of PTC alone (30). We previously found that patients with NG had an obviously higher MUI than healthy controls, and high iodine intake was associated with a larger tumor size of NG (11). We expected that PTMC patients with NG may be treated at an earlier stage compared with patients with PTMC alone. Thus, these patients were more likely to be negative for cervical lymph nodes.

The mutation rate of BRAF^V600E^ in PTC was up to 86.7%, whereas it was not found to be associated with CLNM (31), and LLNM of PTMC (13). However, some reported that BRAF^V600E^ mutation was independently associated with CLNM when PTMC ≤5 mm (32). It was reported that low iodine intake (UIC <300 μg/L) and more than excessive iodine intake (UIC ≥500 μg/L) were associated with BRAF^V600E^ mutation of PTC (33). However, some reported that there were no significant differences in the prevalence of BRAF^V600E^ mutation between PTC patients in iodine-rich areas and those in iodine-deficient areas (34). In addition, some researchers found that the MUI was not significantly different in patients with BRAF^V600E^ mutation or not (21). Therefore, the relationship between iodine nutrition and BRAF^V600E^ should be investigated further.

CLNM was positively associated with LLNM in both male and female PTMC patients, which was consistent with previous reports (24, 25). As far as we know, this is the largest study focusing on the association between iodine nutrition and PTMC progression. However, some shortcomings should be acknowledged. First, the retrospective study nature cannot demonstrate the cause and effect between iodine nutrition and nodal metastasis of PTMC. Second, the iodine nutrition of patients may not be evaluated accurately by a single determination of UIC. Third, we failed to assess the genetic alterations of PTMC. Last but not least, we cannot explore the underlying mechanisms between iodine intake and PTC initiation and progression.

In conclusion, we investigated the association between iodine nutrition and cervical lymph node metastasis of PTMC patients. We found that more than adequate iodine intake was associated with CLNM and extrathyroidal extension compared with adequate iodine intake in female PTMC patients. Iodine nutrition was more closely associated with tumor progression in female patients. The following studies with a larger sample size should be done to further illuminate the association between iodine nutrition and PTC progression.

## Data availability statement

The original contributions presented in the study are included in the article/**Supplementary Material**. Further inquiries can be directed to the corresponding authors.

## Ethics statement

The studies involving humans were approved by Ethics Committee of Union hospital. The studies were conducted in accordance with the local legislation and institutional requirements. Written informed consent for participation was not required from the participants or the participants’ legal guardians/next of kin in accordance with the national legislation and institutional requirements.

## Author contributions

HZ and JH contributed the design of the study and writing of the manuscript. HZ, JH and LC collected the data and performed the analyses. YG and TH supervised the work. All authors reviewed and approved the final version of the manuscript.

## References

[1] Lim H Devesa SS Sosa JA Check D Kitahara CM. Trends in thyroid cancer incidence and mortality in the United States, 1974-2013. JAMA (2017) 317:1338–48. doi: 10.1001/jama.2017.2719 PMC8216772 28362912

[2] Wang J Yu F Shang Y Ping Z Liu L. Thyroid cancer: incidence and mortality trends in China, 2005-2015. Endocrine (2020) 68:163–73. doi: 10.1007/s12020-020-02207-6 32002755

[3] Seib CD Sosa JA. Evolving understanding of the epidemiology of thyroid cancer. Endocrinol Metab Clin North Am (2019) 48:23–35. doi: 10.1016/j.ecl.2018.10.002 30717905

[4] Du L Wang Y Sun X Li H Geng X Ge M. Thyroid cancer: trends in incidence, mortality and clinical-pathological patterns in Zhejiang Province, Southeast China. BMC Cancer (2018) 18:291. doi: 10.1186/s12885-018-4081-7 29544469 PMC5856225

[5] Li Y Teng D Ba J Chen B Du J He L. Efficacy and safety of long-term universal salt iodization on thyroid disorders: epidemiological evidence from 31 provinces of mainland China. Thyroid (2020) 30:568–79. doi: 10.1089/thy.2019.0067 32075540

[6] Zhao H Tian Y Liu Z Li X Feng M Huang T. Correlation between iodine intake and thyroid disorders: a cross-sectional study from the South of China. Biol Trace Elem Res (2014) 162:87–94. doi: 10.1007/s12011-014-0102-9 25161089

[7] Zhang X Zhang F Li Q Feng C Teng W. Iodine nutrition and papillary thyroid cancer. Front Nutr (2022) 9:1022650. doi: 10.3389/fnut.2022.1022650 36337631 PMC9631789

[8] Fan L Meng F Gao Y Liu P. Insufficient iodine nutrition may affect the thyroid cancer incidence in China. Br J Nutr (2021) 126:1852–60. doi: 10.1017/s0007114521000593 33597052

[9] Kim K Cho SW Park YJ Lee KE Lee DW Park SK. Association between iodine intake, thyroid function, and papillary thyroid cancer: A case-control study. Endocrinol Metab (Seoul) (2021) 36:790–99. doi: 10.3803/EnM.2021.1034 PMC8419609 34376043

[10] Inoue K Leung AM Sugiyama T Tsujimoto T Makita N Nangaku M. Urinary iodine concentration and mortality among U.S. Adults. Thyroid (2018) 28:913–20. doi: 10.1089/thy.2018.0034 PMC6916127 29882490

[11] Zhao H Li H Huang T. High urinary iodine, thyroid autoantibodies, and thyroid-stimulating hormone for papillary thyroid cancer risk. Biol Trace Elem Res (2018) 184:317–24. doi: 10.1007/s12011-017-1209-6 29164514

[12] Zhao H Li H Huang T. High iodine intake and central lymph node metastasis risk of papillary thyroid cancer. J Trace Elem Med Biol (2019) 53:16–21. doi: 10.1016/j.jtemb.2019.01.015 30910201

[13] Kim K Zheng X Kim JK Lee CR Kang SW Lee J. The contributing factors for lateral neck lymph node metastasis in papillary thyroid microcarcinoma (PTMC). Endocrine (2020) 69:149–56. doi: 10.1007/s12020-020-02251-2 32146654

[14] Siddiqui S White MG Antic T Grogan RH Angelos P Kaplan EL. Clinical and pathologic predictors of lymph node metastasis and recurrence in papillary thyroid microcarcinoma. Thyroid (2016) 26:807–15. doi: 10.1089/thy.2015.0429 27117842

[15] Zeng Z Li K Wang X Ouyang S Zhang Z Liu Z. Low urinary iodine is a protective factor of central lymph node metastasis in papillary thyroid cancer: a cross-sectional study. World J Surg Oncol (2021) 19:208. doi: 10.1186/s12957-021-02302-6 34253203 PMC8276512

[16] Fan L Tian Q Xiu C Wang F Yuan Z He Q. High iodine nutrition may be a risk factor for cervical lymph node metastasis in papillary thyroid cancer patients. Ann Nutr Metab (2021) 77:90–9. doi: 10.1159/000513334 34289482

[17] Lee JH Hwang Y Song RY Yi JW Yu HW Kim SJ. Relationship between iodine levels and papillary thyroid carcinoma: A systematic review and meta-analysis. Head Neck (2017) 39:1711–18. doi: 10.1002/hed.24797 28513893

[18] Choi JY Lee JH Song Y. Evaluation of iodine status among korean patients with papillary thyroid cancer using dietary and urinary iodine. Endocrinol Metab (Seoul) (2021) 36:607–18. doi: 10.3803/EnM.2021.1005 PMC8258329 34154044

[19] Huang F Cong W Xiao J Zhou Y Gong M Sun J. Association between excessive chronic iodine exposure and the occurrence of papillary thyroid carcinoma. Oncol Lett (2020) 20:189. doi: 10.3892/ol.2020.12051 32952658 PMC7479532

[20] Zhang L Fang C Liu L Liu X Fan S Li J. A case-control study of urinary levels of iodine, perchlorate and thiocyanate and risk of papillary thyroid cancer. Environ Int (2018) 120:388–93. doi: 10.1016/j.envint.2018.08.024 30125856

[21] Lee JH Song RY Yi JW Yu HW Kwon H Kim SJ. Case-control study of papillary thyroid carcinoma on urinary and dietary iodine status in South Korea. World J Surg (2018) 42:1424–31. doi: 10.1007/s00268-017-4287-x 29067516

[22] Yan AR Zhang X Shen H Zhou X Li R Yuan Z. Urinary iodine is increased in papillary thyroid carcinoma but is not altered by regional population iodine intake status: a meta-analysis and implications. Endocr J (2019) 66:497–514. doi: 10.1507/endocrj.EJ18-0532 30890682

[23] Yu Z Yu Y Wan Y Fan J Meng H Li S. Iodine intake level and incidence of thyroid disease in adults in Shaanxi province: a cross-sectional study. Ann Transl Med (2021) 9:1567. doi: 10.21037/atm-21-4928 34790773 PMC8576709

[24] Kim SK Park I Woo JW Lee JH Choe JH Kim JH. Predictive factors for lymph node metastasis in papillary thyroid microcarcinoma. Ann Surg Oncol (2016) 23:2866–73. doi: 10.1245/s10434-016-5225-0 27075321

[25] Sheng L Shi J Han B Lv B Li L Chen B. Predicting factors for central or lateral lymph node metastasis in conventional papillary thyroid microcarcinoma. Am J Surg (2020) 220:334–40. doi: 10.1016/j.amjsurg.2019.11.032 31818425

[26] Xiang J Wang X Wang Z Wu Y Li D Shen Q. Effect of different iodine concentrations on well-differentiated thyroid cancer cell behavior and its inner mechanism. Cell Biochem Biophys (2015) 71:299–305. doi: 10.1007/s12013-014-0198-8 25120024

[27] Teng D Yang W Shi X Li Y Ba J Chen B. An inverse relationship between iodine intake and thyroid antibodies: A national cross-sectional survey in mainland China. Thyroid (2020) 30:1656–65. doi: 10.1089/thy.2020.0037 32586221

[28] Mao A An N Wang J Wu Y Wang T Wang Z. Association between preoperative serum TSH and tumor status in patients with papillary thyroid microcarcinoma. Endocrine (2021) 73:617–24. doi: 10.1007/s12020-021-02690-5 33755880

[29] Hollowell JG Staehling NW Flanders WD Hannon WH Gunter EW Spencer CA. T(4), and thyroid antibodies in the United States population (1988 to 1994): National Health and Nutrition Examination Survey (NHANES III). J Clin Endocrinol Metab (2002) 87:489–99. doi: 10.1210/jcem.87.2.8182 11836274

[30] Huang J Lin C Chen Y Li X. Clinical preliminary study on the correlation between nodular goitre and papillary thyroid carcinoma. Transl Cancer Res (2020) 9:3794–803. doi: 10.21037/tcr-19-2951 PMC8798884 35117747

[31] Li X Li E Du J Wang J Zheng B. BRAF mutation analysis by ARMS-PCR refines thyroid nodule management. Clin Endocrinol (Oxf) (2019) 91:834–41. doi: 10.1111/cen.14079 31441082

[32] Zhou SL Guo YP Zhang L Deng T Xu ZG Ding C. Predicting factors of central lymph node metastasis and BRAF(V600E) mutation in Chinese population with papillary thyroid carcinoma. World J Surg Oncol (2021) 19:211. doi: 10.1186/s12957-021-02326-y 34256769 PMC8278623

[33] Kim HJ Park HK Byun DW Suh K Yoo MH Min YK. Iodine intake as a risk factor for BRAF mutations in papillary thyroid cancer patients from an iodine-replete area. Eur J Nutr (2018) 57:809–15. doi: 10.1007/s00394-016-1370-2 28258306

[34] Vuong HG Kondo T Oishi N Nakazawa T Mochizuki K Inoue T. Genetic alterations of differentiated thyroid carcinoma in iodine-rich and iodine-deficient countries. Cancer Med (2016) 5:1883–9. doi: 10.1002/cam4.781 PMC4898973 27264674

