# Supplementary material for: Association between iodine nutrition and cervical lymph node metastasis of papillary thyroid microcarcinoma
Source: Front Endocrinol (Lausanne). 2023 Aug 31;14:1164069. doi: 10.3389/fendo.2023.1164069 (PMC10500345; doi:10.3389/fendo.2023.1164069)
Supplement: Supplementary file 1 [file Table_1.docx]

Supplementary Material

| **Supplementary table 1.** The iodine nutrition between PTMC patients with lymph node metastasis and non-lymph node metastasis. | | | |
| --- | --- | --- | --- |
| Category |  |  | *P*-value |
| Iodine nutrition (μg/L) | Non-CLNM | CLNM |  |
| ≤ 99.9 | 450 (19.2) | 279 (18.4) | 0.366 |
| 100.0-199.9 | 941 (40.2) | 589 (38.8) |  |
| 200.0-299.9 | 551 (23.5) | 395 (26.0) |  |
| ≥ 300.0 | 399 (17.0) | 254 (16.7) |  |
|  | Non-LLNM | LLNM |  |
| ≤ 99.9 | 684 (18.9) | 45 (19.1) | 0.683 |
| 100.0-199.9 | 1429 (39.4) | 101 (43.0) |  |
| 200.0-299.9 | 894 (24.7) | 52 (22.1) |  |
| ≥ 300.0 | 616 (17.0) | 37 (15.7) |  |
|  | Male | Female |  |
| ≤ 99.9 | 87 (13.4) | 642 (20.0) | 0.001 |
| 100.0-199.9 | 283 (43.5) | 1247 (38.9) |  |
| 200.0-299.9 | 173 (26.6) | 773 (24.1) |  |
| ≥ 300.0 | 107 (16.5) | 546 (17.0) |  |
|  | Non-extrathyroidal extension | Extrathyroidal extension |  |
| ≤ 99.9 | 694 (19.2) | 35 (14.7) | 0.029 |
| 100.0-199.9 | 1446 (39.9) | 84 (35.3) |  |
| 200.0-299.9 | 871 (24.1) | 75 (31.5) |  |
| ≥ 300.0 | 609 (16.8) | 44 (18.5) |  |
| PTMC, papillary thyroid microcarcinoma; CLNM, central lymph node metastasis; LLNM, lateral lymph node metastasis. | | | |

| **Supplementary table 2.** Predictors for extrathyroidal extension in female PTMC patients. | | | | |
| --- | --- | --- | --- | --- |
| Variables | Univariate | | Multivariate | |
|  | OR (95% CI) | *P* | OR (95% CI) | *P* |
| Age (year) |  |  |  |  |
| < 55 | Ref |  | Ref |  |
| ≥ 55 | 1.31 (0.91-1.87) | 0.148 | 1.54 (1.08-2.18) | 0.030 |
| Tumor size (cm) |  |  |  |  |
| D ≤ 0.5 | Ref |  | Ref |  |
| 0.5 < D ≤ 1.0 | 3.05 (2.19-4.23) | < 0.001 | 1.54 (1.08-2.18) | 0.016 |
| Tumor number |  |  |  |  |
| Solitary | Ref |  | Ref |  |
| Multiple | 1.69 (1.26-2.28) | 0.001 | 1.25 (0.78-1.99) | 0.355 |
| Bilaterality |  |  |  |  |
| No | Ref |  | Ref |  |
| Yes | 1.65 (1.22-2.24) | 0.001 | 0.97 (0.60-1.56) | 0.896 |
| Capsular invasion | |  |  |  |
| No | Ref |  | Ref |  |
| Yes | 65.47 (20.87-205.33) | < 0.001 | 52.97 (16.80-167.03) | < 0.001 |
| Intrathyroidal spread | |  |  |  |
| No | Ref |  | Ref |  |
| Yes | 1.66 (0.79-3.49) | 0.180 | 0.64 (0.29-1.40) | 0.261 |
| CLNM |  |  |  |  |
| No | Ref |  | Ref |  |
| Yes | 1.91 (1.42-2.57) | < 0.001 | 1.12 (0.80-1.56) | 0.519 |
| LLNM |  |  |  |  |
| No | Ref |  | Ref |  |
| Yes | 3.64 (2.37-5.59) | < 0.001 | 2.38 (1.47-3.87) | < 0.001 |
| Pathology |  |  |  |  |
| PTMC | Ref |  | Ref |  |
| PTMC&NG&HT | 0.56 (0.34-0.92) | 0.023 | 0.65 (0.38-1.11) | 0.112 |
| PTMC&HT | 0.69 (0.48-0.99) | 0.041 | 0.66 (0.45-0.96) | 0.029 |
| PTMC&NG | 0.67 (0.43-1.04) | 0.077 | 0.73 (0.46-1.17) | 0.191 |
| UIC (μg/L) | |  |  |  |
| ≤ 99.9 | 0.84 (0.53-1.33) | 0.457 | 0.89 (0.55-1.43) | 0.620 |
| 100.0-199.9 | Ref |  | Ref |  |
| 200.0-299.9 | 1.70 (1.18-2.44) | 0.004 | 1.59 (1.09-2.32) | 0.017 |
| ≥ 300.0 | 1.23 (0.80-1.90) | 0.352 | 1.26 (0.80-1.99) | 0.316 |
| LLNM, lateral lymph node metastasis; PTMC, papillary thyroid microcarcinoma; OR (95% CI), odds ratio (95% confidence interval); D, diameter; CLNM, central lymph node metastasis; HT, Hashimoto's thyroiditis; NG, nodular goiter; UIC, urinary iodine concentration. | | | | |
